# Family conferences and shared prioritisation to improve patient safety in the frail elderly (COFRAIL): study protocol of a cluster randomised intervention trial in primary care

**DOI:** 10.1186/s13063-020-4182-x

**Published:** 2020-03-20

**Authors:** Achim Mortsiefer, Stefan Wilm, Sara Santos, Susanne Löscher, Anja Wollny, Eva Drewelow, Manuela Ritzke, Petra Thürmann, Nina-Kristin Mann, Gabriele Meyer, Jens Abraham, Andrea Icks, Joseph Montalbo, Birgitt Wiese, Attila Altiner, on behalf of the COFRAIL study group, Johanna Bensch, Madaida Lemke, Henrik Wiegelmann, Marjan van den Akker, Christoph Ostgathe, Vera Kalitzkus, Sven Schmiedl, Veronika Bencheva, Steffen Fleischer, Markus Vomhof

**Affiliations:** 1 Institute of General Practice, Medical Faculty, Heinrich-Heine-University, Moorenstr. 5, 40225 Düsseldorf, Germany; 2 Institute of General Practice, University Medical Center Rostock, Doberaner Str. 142, 18057 Rostock, Germany; 3 Department of Clinical Pharmacology, School of Medicine, Faculty of Health, Witten/Herdecke University, Heusnerstr. 40, 42283 Wuppertal, Germany; 4 Institute for Health and Nursing Science, Medical Faculty, Martin Luther University Halle-Wittenberg, Magdeburger Str. 8, 06112 Halle (Saale), Germany; 5 Institute for Health Services and Economics, Centre for Health and Society, Faculty of Medicine, Heinrich-Heine-University Düsseldorf, Moorenstr. 5, 40225 Düsseldorf, Germany; 6 WG Medical Statistics and IT-Infrastructure, Institute of General Practice, Hannover Medical School, Carl-Neuberg-Str. 1, 30625 Hannover, Germany

**Keywords:** Frailty, Elderly patients, Polypharmacy, Family conferences, Primary care, Deprescribing, Shared decision making, Cluster randomised controlled trial, Study protocol

## Abstract

**Background:**

Frailty in elderly patients is associated with an increased risk of poor health outcomes, including falls, delirium, malnutrition, hospitalisation, and mortality. Because polypharmacy is recognised as a possible major contributor to the pathogenesis of geriatric frailty, reducing inappropriate medication exposure is supposed to be a promising approach to improve health-related quality of life and prevent adverse outcomes. A major challenge for the process of deprescribing of inappropriate polypharmacy is to improve the communication between general practitioner (GPs), patient and family carer. This study investigates the effects of a complex intervention in frail elderly patients with polypharmacy living at home.

**Methods:**

This is a cluster randomised controlled trial including 136 GPs and 676 patients. Patients with a positive clinical screening for frailty are eligible if they are aged 70 years or older, receiving family or professional nursing care at home, and taking in five or more drugs per day. Exclusion criteria are higher grade of dementia and life expectancy of 6 months or less. The GPs of the intervention group receive an educational training promoting a deprescribing guideline and providing information on how to conduct a family conference focussing on prioritisation of treatment goals concerning drug therapy. During the 1-year intervention, GPs are expected to perform a total of three family conferences, each including a structured medication review with patients and their family carers. GPs of the control group will receive no training and will deliver care as usual. Geriatric assessment of all patients will be performed by study nurses during home visits at baseline and after 6 and 12 months. The primary outcome is the hospitalisation rate during the observation period of 12 months. Secondary outcomes are number and appropriateness of medications, mobility, weakness, cognition, depressive disorder, health-related quality of life, activities of daily living, weight, and costs of health care use.

**Discussion:**

This study will provide evidence for a pragmatic co-operative and patient-centred educational intervention using family conferences to improve patient safety in frail elderly patients with polypharmacy.

**Trial registration:**

German Clinical Trials Register, DRKS00015055 (WHO International Clinical Trials Registry Platform [ICTRP]). Registered on 6 February 2019.

## Background

Frailty in elderly patients is a vulnerable health status characterised by an increased risk of adverse health outcomes and/or dying when exposed to a stressor [1, 2]. Physical frailty is defined as a clinical syndrome or multidimensional risk state often associated with weight loss, sarcopenia, weakness, exhaustion, and reduced physical activity [3, 4]. Frailty is associated with an increased risk of poor health outcomes, including falls, delirium, malnutrition, hospitalisation, and mortality [1, 5]. However, geriatric frailty is not a categorical irreversible status; it can be addressed by various interventions [6, 7].

Polypharmacy is recognised as a possible major contributor to the pathogenesis of frailty [8]. Despite the undisputable benefit of drug treatment for certain conditions, a large number of medications in patients with multimorbidity increases the risk of adverse drug reactions (ADRs) due to age-related changes in pharmacokinetics, pharmacodynamics, and physiology (resulting in higher sensitivity), as well as due to drug interactions [9, 10]. ADRs are found in 35% of older people cared for at home [11]. It has been assumed that 2.4–6.5% of all hospital admissions are drug related. In older people, these figures are considerably higher [12–14]. Polypharmacy independently increases the risk of falling, frailty, hospital admission [15–17], and mortality [18]. Excessive polypharmacy (ten or more drugs) is an independent risk factor for death in vulnerable geriatric patients [18]. Reducing inappropriate medication exposure in frail patients is supposed to be a promising approach to improve health-related quality of life and prevent adverse outcomes [19–21].

Only very few randomised controlled trials have been conducted to investigate the outcomes of medication tapering or withdrawal of single drugs such as antihypertensives, statins, or benzodiazepines [22]. Particularly, the reduction of psychotropic drugs can often be realised with beneficial effects for patients [23]. In frail patients, drug treatment aimed at prolonging life (e.g., cardiovascular disease prevention) is often a less important therapeutic goal due to limited overall life expectancy [18], and a significant proportion of older adults is willing to reduce their number of daily drugs [24].

Selecting appropriate patients on the basis of fully informed consent from patients and carers and taking a palliative care perspective with the intention to reduce polypharmacy has been shown to be a successful process for improving older persons’ quality of life [25]. A recent review summarised the effects of interventions on optimising polypharmacy in older people and found that most of the interventions were aimed at optimising surrogate outcomes such as number of drugs and number of inappropriate medications. However, the impact on patient-relevant outcomes has not been properly studied yet [26].

Many barriers to deprescribing inappropriate polypharmacy have been identified in physicians, patients, and relatives, resulting in major challenges to the communication process between the participating actors [24, 27]. Therefore, interventions to reduce polypharmacy should be embedded in a shared decision-making process of general practitioners (GPs), patients, and family carers [28]. Family carers play a pivotal role in daily drug management, such as by avoiding failure to receive drugs, paying attention to ADRs, and supporting patient adherence [29].

Family conferences are a well-established instrument in nursing, intensive care units, and palliative care [30, 31]. Family conferences are a forum for communication between patients, physicians, nursing staff, and family members [31–33]. From the families’ point of view, treatment changes for a chronic illness are among the three major indications for a family conference with a GP (apart from terminal illness and hospitalisation). Although family conferences are effective in improving the communication process [31] and involvement of frail patients in planning conferences is possible [34], up to now, no intervention study has investigated their effects on polypharmacy in frail patients cared for at home.

This study evaluates the effects of a complex intervention including repeated structured medication reviews and family conferences in frail elderly patients with polypharmacy living at home. The primary hypothesis is that patient safety operationalised as the hospitalisation rate will be reduced in the intervention group compared with the control group with usual care within the study period of 12 months. Secondary hypotheses include that the intervention will reduce the number of prescribed inappropriate drugs, improve health-related quality of life, reduce the rate of falls, and reduce the rate of emergency services. Additionally, we expect that in the intervention group, self-determination of patients will improve and the process of shared decision making between GPs, patients, and family carers will be enhanced.

## Methods

### Study design

A cluster randomised controlled trial will be performed to assess the effectiveness of a single-arm complex intervention for GPs, their geriatric patients with frailty and polypharmacy living at home, and the patients’ family carers. Cluster randomisation at the level of practices/medical centres is necessary to avoid contamination because the intervention addresses a change of the professional performance of the GPs. The primary endpoint (hospitalisation rate) is measured at the patient level. Intervention/observation time per patient will be 12 months. Time between first patient in and last patient out will be approximately 15 months per GP practice and 18 months for the complete trial. The design is open label with only outcome assessors being blinded. The Consolidated Standards of Reporting Trials (CONSORT) statement (with extension for cluster trials) has been used to design the study and will be used when reporting the results.

### Study setting and participants

**Fig. 1:**
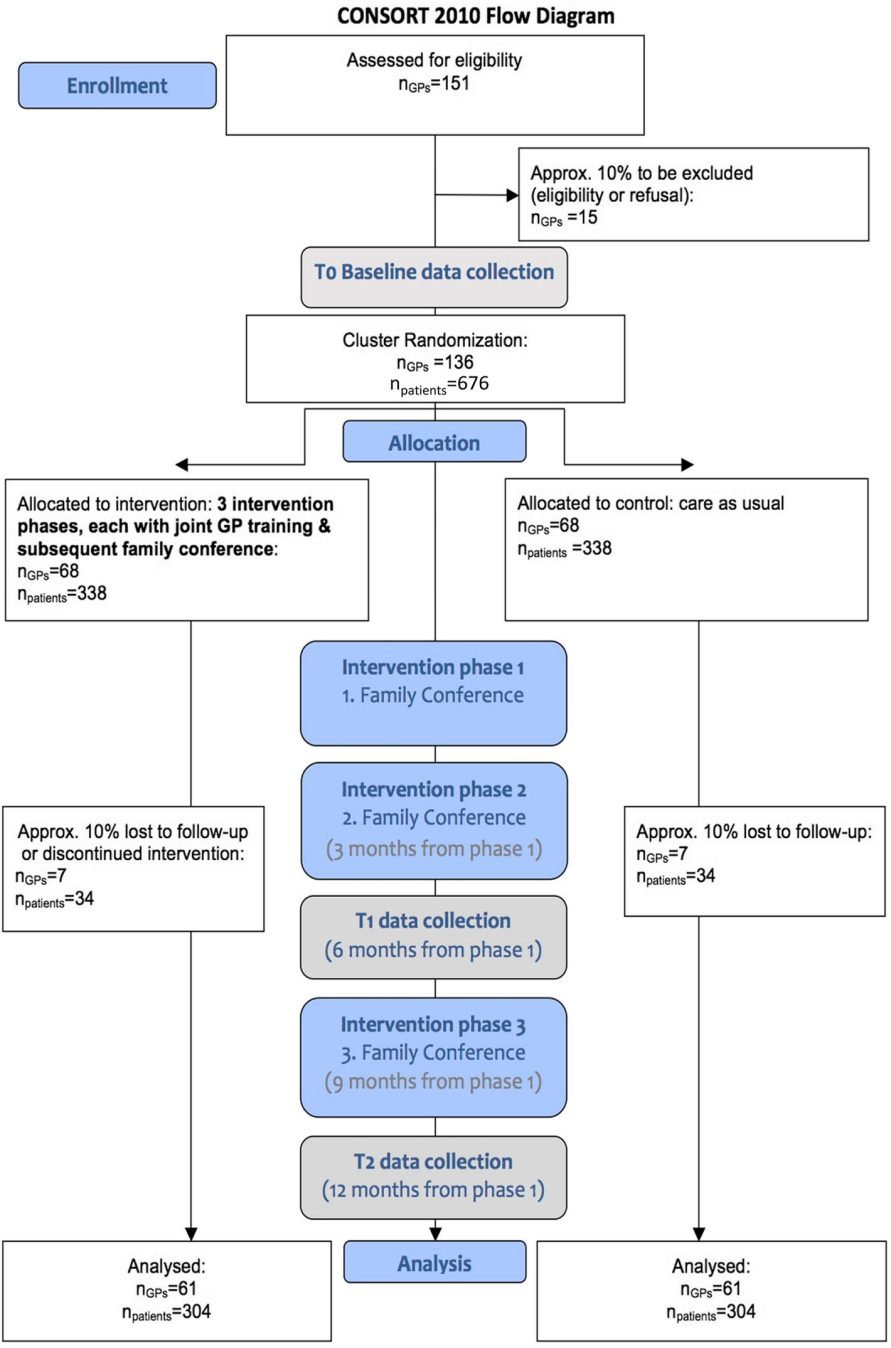
Family Conferences and Shared Prioritization to Improve Patient Safety in the Frail Elderly (COFRAIL) study flow chart

This study will be performed in a primary care setting in two areas of Germany (Düsseldorf and Rostock). At first, 136 GPs will be recruited. The GPs will be requested to enrol 676 frail elderly patients (average of 5 patients per GP) with polypharmacy living at home (Fig. 1). Patients are eligible to participate in the study if they meet all of the following criteria:

1. Positive screening of frailty in a short screening questionnaire answered by their GP using the Canadian Study of Health and Aging Clinical Frailty Scale [5]. Patients at levels 5–7 are eligible to participate in this trial.
2. Aged 70 years or older
3. Regular intake of five or more different drugs per day (defined as polypharmacy)
4. Care dependency (need for care assessed by the medical service of the German long-term care insurance) or comparable status
5. Receiving nursing care in the domestic environment, provided either by informal family carers alone or by professional ambulatory care services

Patients are not eligible to participate in the study if they meet one or more of the following criteria as rated by their GPs:

1. Moderate or severe dementia
2. Being under legal guardianship
3. Reduced life expectancy of 6 months or less (palliative care)
4. Living in a nursing home
5. Insufficient German-language skills of patients and/or family carer and no translator available

### Recruitment and incentives

The study centres will send an invitation letter to the local GPs with a request to answer by email or fax if interested in participating in the study. In the next step, the GPs will be contacted by the study staff in order to hand out further information and to get informed consent of the GPs. In addition, some local information events on the topic of geriatric frailty syndrome will be offered to all interested GPs without disclosing the study objective, which is deprescribing. Each of the enrolled GPs is requested to include five eligible patients in the study.

Participating GPs in both the intervention group and the control group receive an expense allowance for every enrolled patient of €100. Patients and carers will not receive financial incentives for participating in the study.

### Intervention

#### Experimental intervention

In the first phase of the Family Conferences and Shared Prioritisation to Improve Patient Safety in the Frail Elderly (COFRAIL) study, a systematic process of intervention development was conducted with collaboration of all project partners. The feasibility of the recruitment process and the COFRAIL intervention was tested in a pilot study. For this purpose, four GPs conducted eight family conferences with frail elderly patients and at least one family member. For evaluation purposes, the GPs and the family carers were interviewed by telephone after the family conferences. Conclusions from the pilot study regarding the educational concept will be drawn by consulting all project partners.

**Fig. 2:**
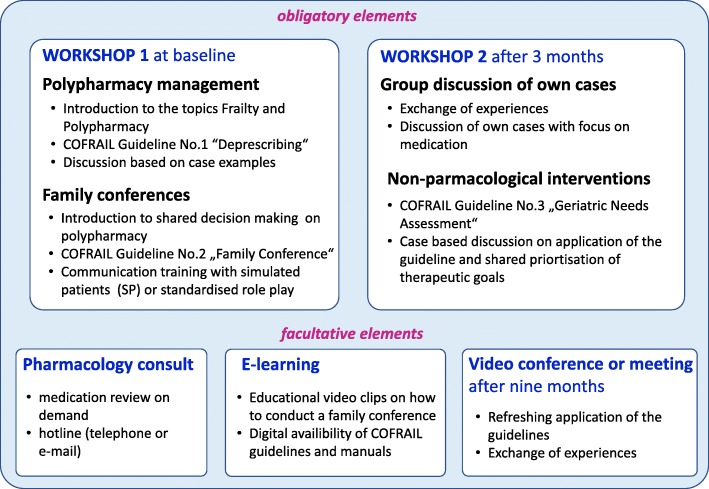
Elements of the educational intervention

The experimental intervention will be performed in two steps:

1. *Step 1*: GPs allocated to the intervention arm will receive an educational intervention covering the following topics: (a) structured patient-centred medication review, (b) family conference according to a structured guideline with special focus on prioritisation of treatment goals concerning drug therapy (communication training included), and (c) structured deprescribing protocol in case of a decision in favour of reducing the drug burden. Over the course of the trial, altogether three trainings (two meetings in the first year and one optional meeting in the second year) are required to achieve the educational objectives and to enhance the safety of the intervention by giving the GPs the opportunity to discuss their experiences with colleagues based on their own cases. An overview of the educational intervention programme is presented in Fig. 2.
2. *Step 2*: During the 1-year intervention, GPs will perform a total of three family conferences per enrolled patient each, including a structured medication review with the enrolled patient and the patient’s family carer instead of routine home visits or consultations. The family conferences are scheduled at the beginning of the study, after 3 months, and after 9 months, each lasting about 30–45 min. The conversations will initially focus on the medications taken by the patient (brown bag review), followed by a discussion about treatment goals and priorities of the patient and the family carers. The family conferences can be conducted optionally in the doctor’s office or at the patient’s home. If desired by the team and/or physician, counselling by an external clinical pharmacologist/pharmacist will be provided via phone or email.

If both patient and physician in agreement consider reducing polypharmacy, the GP is recommended to follow a structured deprescribing protocol based on those published by Scott [25] and Garfinkel [20], including five steps: (1) ascertain all drugs the patient is currently taking and the reasons for each one, (2) consider overall risk of drug-induced harm in individual patients to determine the required intensity of deprescribing intervention, (3) assess each drug in regard to its current or future benefit compared with current or future harm or potential burden, (4) prioritise drugs for discontinuation that have the lowest benefit-to-harm ratio and lowest likelihood of adverse withdrawal reactions or disease rebound syndromes, and (5) implement a discontinuation regime and monitor patients closely for improvement in outcomes or onset of adverse effects.

In addition, the GP has the option to obtain a written medication review for the patient by a clinical pharmacologist associated with the study group.

There will be no special criteria for discontinuing or modifying allocated interventions. Implementing training and up to three family conferences may alter usual care pathways (including use of any medication) in GP practices randomised to the intervention.

There is no anticipated harm and no compensation for trial participation. It is not necessary to provide post-trial care.

#### Control group

Patients in the control group will receive care as usual. GPs in the control group will be offered training seminars on clinical topics for daily practice that do not interfere with the intervention.

At the end of the observational period, the educational intervention will be offered to GPs in the control group due to motivational reasons. This offer will not influence study outcomes but should maintain the motivation of the GPs assigned randomly to the control arm.

### Measurements

At the beginning of the trial, the following parameters will be collected on the GP’s level by a self-administered questionnaire: age, sex, number of practicing GPs in the practice, number of treatment cases per year, year of licensure to practice medicine, and board-certified specialities. The GPs will be requested to provide the following data of patients from the patients’ medical records at baseline: chronic diagnoses and medication schedule. After 6 and 12 months, new chronic diagnoses and medication schedules shall be reported. This trial does not involve collecting biological specimens.

A geriatric assessment of patients will be performed by trained study nurses during home visits at baseline, after 6 months (T_1_), and after 12 months (T_2_). The following sociodemographic parameters of patients will be recorded by study nurses: age, sex, level of education, marital status, household members, degree of disability (*Grad der Behinderung*), level of care dependency assessed by the medical service of the German social care insurance (*Pflegegrad*). In addition, the study nurse will collect data on all medications used by the patient within the last week. The data include product name, pharmaceutical form, content of the active substance, German national drug code, periodic or as needed (*pro re nata*) medication, dosage and frequency (for periodic medication), and duration of prescription (less than 1 week, 1 week or longer but less than 1 month, 1 month or longer but less than 2 months, 2 months or longer but less than 6 months, 6 months or longer but less than 1 year, or 1 year or longer). The interviewer will ask the patient to show the packages of the pharmaceuticals to get the most valid information.

The patients will be requested to keep an event diary to document number of falls, weight, hospitalisations, and any other health care use which is further explained below under the heading ‘Additional health economic parameters’. The event diary will be handed over, collected, and validated by the study nurses during their home visits.

#### Primary outcome

The primary endpoint of the study is the number of hospitalisations per patient during the observation period of 12 months. This outcome does not include visits to an outpatient clinic or visits to an emergency unit without hospital admission. The hospitalisations will be documented by the participating GPs after 6 and 12 months and by the patients in their event diary. In case of disparity between the number of hospitalisations documented by GPs and patients, the study centre will be requested to validate the information on hospitalisation. If the information cannot be clarified, the higher value will be used for data analysis.

### Secondary outcomes

The following secondary outcomes will be analysed on the basis of data collected by study nurses during home visits at baseline and after 6 and 12 months:

- Total number of medications, Drug Burden Index defined as the number of anticholinergic and sedative medications [16], prevalence of potentially inappropriate medications (PIMs) [35, 36], medication regimen complexity [37], and drug–drug interactions
- Weakness measured by grip strength using a dynamometer (model SH5001; Saehan Corp., Masan, Republic of Korea); best of three attempts used
- Cognition: Two short cognitive subtests from the Consortium to Establish a Registry for Alzheimer’s Disease neuropsychological test battery, German version, with norms and reliable change indices recently derived from an older German GP sample [38, 39]:
  - ◦ Episodic memory: word list learning (three trials immediate and one trial delayed recall); total administration time: maximum of 10 min plus 10-min interval and approximately 30 s for instruction
  - ◦ Executive function: semantic fluency (animal naming test) as a measure of combined verbal ability and executive control; administration time 1 min plus approximately 20 s for instruction
- Depressive disorder assessed by the Geriatric Depression Scale [40]
- Mobility measured by the Timed Up & Go Test [41]
- Health-related quality of life (EQ-5D-5L) will be assessed to facilitate calculation of quality-adjusted life-years (QALYs) for use in the economic analysis [42].
- Activities of daily living measured by the Barthel index [43]
- Weight (self-reported)
- Number of falls (self-reported)
- Use of emergency services

Detailed procedures of the measurements are listed in the schedule of enrolment, interventions, and assessments (Table 1).

**Table 1:** Adapted SPIRIT schedule of enrolment, interventions, and assessments

	Study period						
	Enrolment	Baseline	Intervention				Close-out
Time point	**− t**_**1**_	**t**_**0**_			**t**_**1**_		**t**_**2**_
Month	− 3	0	1	3	6	9	12
Enrolment							
Eligibility screen	X						
Informed consent	X						
Allocation		X					
Interventions							
Intervention group (family conferences)			X	X		X	
Control group (care as usual)							
Assessments							
Hospitalisation rate (NHPP)					X		X
Medication (TNM, DBI, PIM)		X			X		X
Grip strength		X			X		X
Cognition (CERAD)		X			X		X
Depression (GDS)		X			X		X
Mobility (Timed Up & Go Test)		X			X		X
Health-related quality of life (EQ-5D-5L)		X			X		X
Activities of daily living		X			X		X
Weight		X			X		X
Number of falls		X			X		X
Use of emergency services		X			X		X

*Abbreviations: CERAD* Consortium to Establish a Registry for Alzheimer’s Disease, *DBI* Drug Burden Index, *GDS* Geriatric Depression Scale, *NHPP* number of hospitalisations per patient, *PIM* potentially inappropriate medications, *SPIRIT* Standard Protocol Items: Recommendations for Interventional Trials, *TNM* total number of medications

Participant retention and complete follow-up will be promoted via phone calls by the study nurses. For patients who drop out of the study, the following parameters are requested by the GPs: hospitalisations, cardiovascular events (e.g., stroke, myocardial infarction), mortality, and other adverse events (AEs).

#### Additional safety parameters

The GPs will be requested to document cardiovascular events (e.g., stroke, myocardial infarction) and mortality after 6 months (T_1_) and after 12 months (T_2_). The following safety parameters, if available, will be collected every 3 months by the GPs from the records:

- Blood pressure level (mmHg)
- Heart rate (beats/min)
- Laboratory results: blood sugar (mg/dl), estimated glomerular filtration rate

#### Additional health economic parameters

- Intervention costs will be collected by the study centres and include personnel expenses, costs for the educational intervention (room and catering) material costs, printing costs, and postage related to manuals for physicians and information brochures for patients
- Costs resulting from the consumption of health-related goods and services due to outpatient visits, visits to other health service providers, emergency admissions, hospital admissions, admissions to rehabilitation facilities, medical appliances, and support in households, and services of the long-term care insurance are estimated on the basis of patient questionnaires

### Sample size

In a similar trial population, the mean rate of hospitalisations amounted to 0.38 per patient with a standard deviation of 0.75 during 6 months [44]. We assume that during an observation period of 12 months, the mean number of hospitalisations per patient will be 0.75 with a common standard deviation of 1.0, and we expect a decrease to a mean number of 0.5 in the intervention group. With a power of 80% and an alpha error of 0.05, we would need a sample size of 253 in each group (two-sided *t* test for equal variances) to detect the expected difference. For a cluster randomised intervention trial, the estimated sample size must be adjusted by the design effect. If an intra-cluster correlation of 0.05 is assumed and about five patients per GP are planned, the design effect would be 1.2 and the sample size would be 608 patients in 122 GP practices. With an estimated drop-out rate of 10% during the study period, 676 patients and 136 GPs will be needed in total.

### Randomisation

A cluster randomisation allocating the GP practices to the intervention or control group will be performed. To ensure a balance in sample size across groups over time, block randomisation of GP practices will be used. The randomisation procedure will be provided by the data management group at Hannover Medical School after enrolment of patients. The allocation sequence is computer-generated and concealed from researchers and interviewers. Randomisation lists will be kept closed. To assure concealment of allocation, no practice can start the intervention until recruitment of patients is complete and randomisation has been performed.

### Data management

Data will be entered in the local centres via an Internet-based electronic data capture system which complies with US Food and Drug Administration requirements (21 CFR part 11) and the guidelines of good clinical practice. The data will be stored in a centralised Oracle database (Oracle Corp., Redwood City, CA, USA). The data will be transferred via 128-bit SSL (Secure Sockets Layer) encryption; there will be no local storage of data. The access to the database and webserver is controlled by two consecutive firewall systems. Data will be stored with a pseudonym. The members of the study teams will have access to the electronic data entry system according to a detailed concept of roles and rights. An audit trail ensures an automatic protocol of all data entries, changes, and deletions.

Contact details (e.g., address, telephone number) of the enrolled GPs and patients will be stored separately in electronic files at the study centres in Rostock and Düsseldorf with secure access for the study staff.

### Data analysis

#### Statistical analyses

The analysis of the primary endpoint will be conducted according to the intention-to-treat principle. Sensitivity analyses will be performed on the basis of the per-protocol population (i.e., all patients who have finished the study without any protocol violations will be included in these analyses). Missing values will not be imputed *a priori*. To evaluate a possible bias due to values that are not randomly missing, sensitivity analyses based on multiple imputation will be performed.

Due to cluster randomisation, hierarchic multilevel models (mixed models) with GP practice as a random effect will be applied for the analyses of primary and secondary endpoints. Possible baseline imbalances and confounding variables (e.g., age, sex, co-morbidities) will be controlled by adjustment.

For the evaluation of the primary endpoint, a linear mixed model analysis of variance will be applied.

The analyses of the secondary endpoints will be performed by using linear mixed models or mixed logistic regression models (binary or ordinal), depending of the underlying distribution of the outcome variable.

#### Health economic evaluation

The objective of the health economic evaluation is to determine the efficacy of the intervention by comparing cost and outcome of the intervention group with cost and outcome of the control group (care as usual). All costs associated with the intervention as well as costs resulting from the consumption of health-related goods and services [45] will be considered from the perspective of the German social insurance (statutory health insurance, long-term care insurance, and pension insurance).

In order to determine the efficacy of the intervention, a cost-effectiveness analysis in terms of additional costs per additional hospital admission averted and a cost–utility analysis, which aims to calculate the additional costs required for an improvement in QALYs, will be performed. While the former yields the incremental cost-effectiveness ratio (ICER), the latter estimates the incremental cost-utility ratio (ICUR). The QALYs are based on health-related quality of life, which will be measured by the EQ-5D-5L and evaluated by a German tariff [46] to generate utilities. ICER and ICUR are calculated similarly as the ratio of the difference in mean costs and difference in mean outcomes between intervention and control group. Both costs and outcomes will be derived from cluster averages.

Statistical analyses will be based on the intention-to-treat principle. A 95% confidence interval will be obtained parametrically for the ICER/ICUR and non-parametrically by a bootstrap procedure [47, 48]. Univariate and probabilistic sensitivity analyses will be performed to estimate the robustness of the ICER/ICUR.

### Process evaluation

To understand the change process by implementation of the intervention, a comprehensive assessment of process measures alongside the experimental study is indispensable [49]. The process evaluation follows international recommendations for process evaluation of complex interventions [50]. Therefore, different process parameters will be assessed on cluster and individual levels, respectively, with qualitative and quantitative methodological approaches (Table 2).

**Table 2:** Elements of the process evaluation

Focus	Documentation/assessment	Measurement point
Feasibility of the intervention	Piloting of family conferences with two physicians/region: **semi-structured telephone interview** with physicians**; semi-structured telephone interview** with patients and relatives	Piloting, prior T_0_
Recruitment procedure of physicians and patients	**Protocol**/region	T_0_
Reasons for non-participation or drop-out	**Structured inquiry and documentation** of reasons	T_0_–T_2_
Description of crucial structure- and process-related factors (CRF) on cluster and patient level	**CRF-baseline data**/cluster and patient	T_0_
Conveyance of the intervention	Mandatory educational sessions: **structured protocol** of each educational session. Use of facultative educational session: **standardised documentation** Use of individual medication reviews in intervention and control group: **standardised documentation**	T_0_ (immediately after the educational intervention) T_0_ T_0_–T_2_
Evaluation of telephone study monitoring of physicians and patients	**Structured protocol**	T_0_–T_2_
Evaluation of training	All participants of the education programme: **Standardised questionnaire** → evaluation of the programme • Attitudes • Acceptance • Self-efficacy • Expectations	T_0_ (after the second education sessions)
Application of training content	All physicians: **semi-structured protocols** evaluation of family conferences • Acceptance • Contents • Duration • Practicability • Need for change	T_0_, after 3 and 9 months (immediately after family conferences)
Experiences of physicians (e.g., attitudes regarding intervention; changes in physician–patient communication; barriers and facilitators)	**Four focus groups**: two/region with 6 to 12 physicians **Guideline-based telephone interviews**: convenience sample of ten physicians/region	T_2_ T_2_
Experiences of families (e.g., consideration of preferences; changes in physician–patient communication; barriers and facilitators)	**Guideline-based telephone interviews**: ten patient–relative dyads/region	After 9 months (immediately after the last family conference)

The recruitment procedure of clusters and patients will be documented, including documentation of the information provided on reasons for non-participation or drop-out. Contextual aspects (e.g., socioeconomic and sociodemographic characteristics of physicians and patients) will be assessed at baseline.

Intervention fidelity will be determined by structured documentation for each education session by the trainers. All participants of the obligatory education sessions will be asked to complete a standardised questionnaire after the second session to evaluate the education programme. The use of the facultative educational elements will be also documented. In addition, telephone study monitoring of physicians and patients will be documented using a structured protocol.

Attitudes and experiences of the physicians related to the COFRAIL intervention, including barriers and facilitators, will be explored through four focus group interviews upon completion of the trial with a convenience sample of 6–12 participants per group. Furthermore, guideline-based telephone interviews will be conducted in a convenience sample of ten physicians per region.

In a subgroup of 20 patient–relative dyads, experiences of families (e.g., consideration of preferences, changes in physician–patient communication, barriers and facilitators) will be assessed after 9 months (immediately after the last family conference) with separate semi-structured telephone interviews.

In addition, medication changes will be analysed for the whole study group, and differences between intervention and control groups will be compared descriptively. A detailed analysis will be performed in a subset of patients to elucidate which recommendations of the deprescribing guideline could be followed and which recommendations were either ignored or proved to be inappropriate. This procedure will allow improvement of the applicability of the guideline.

The process evaluation is purely exploratory. All quantitative data will be analysed descriptively, and the qualitative data will be analysed by content analysis [51] (Table 2).

### Quality assurance and safety

Process flow and quality management of the study will be supervised quarterly by a scientific advisory board in repeated audits with study staff. The scientific advisory board will be involved in all decisions on important protocol modifications (e.g., changes to eligibility criteria, outcomes, analyses), followed by informing the relevant parties (investigators, trial participants, trial registries, journals, regulators).

During the intervention, each GP in the intervention group will be contacted at least twice by telephone to evaluate the progress of the study and to get information about potential harms for the patients regarding the safety parameters. Evidence suggests that serious AEs such as cardiovascular events (myocardial infarction, stroke) or death are not anticipated. Potential minor AEs are hyperglycaemia, elevated blood pressure, and recurrence of symptoms (e.g., dyspnoea, oedema) after stopping medication. The events will be reported to the data and safety monitoring board (DSMB) and relevant regulatory bodies as required, indicating expectedness, seriousness, severity, and causality.

To ensure high data quality, assessors (study nurses) will be trained on standardised patients regarding how to interview the patients and how to use the assessment tools. The personnel of the participating practices will not be involved in the collection of data.

Reliability training and checks will be performed before starting the study with the whole staff involved in interviewing and data collection. The quality assurance consists of procedures for prevention of insufficient data quality, detection of inaccurate or incomplete data, and action to improve data quality. In addition, the centres will regularly receive feedback by quality reports for data quality. External monitoring (e.g., for source data verification) would be desirable but too cost-intensive. As a solution, a random sample of paper crucial structure- and process-related factors will be compared with the data entries in the database.

For supervision of the study concerning the safety parameters and safety rules, a DSMB will be constituted. The DSMB will meet at least every 3 months starting and will evaluate the continuously collected safety parameters in a quarterly safety report. For this panel, at least two clinical and/or pharmacological researchers will be recruited who are not part of the COFRAIL project team or advisory board.

If the hospitalisation rate in the intervention group or in the control group will exceed more than 100% in comparison to the other group, the DSMB will inform the primary investigator. The primary investigator shall be responsible to consider further action to assure the safety of the study patients in consensus with all project partners and the DSMB.

### Dissemination policy

The results of the study will be published in brief reports on the website of the project and in user-friendly journals for GPs and other health professionals. For scientific dissemination, the study results will be presented at national and international scientific conferences on health care research, general practice, and clinical pharmacology. We will publish the obtained scientific findings of the trial preferably in open-access journals.

## Discussion

This study will provide evidence for a co-operative and patient-centred educational intervention using family conferences to improve patient safety in frail elderly patients with polypharmacy.

To our knowledge, this is the first randomised controlled trial aiming to assess the effects of family conferences on the safety of patients with geriatric frailty and polypharmacy. The intention of this project is to contribute to the complex process of deprescribing that is often characterised by vague fears of legal consequences and unclear responsibilities of the encountered actors. The strength of this study is that it will be conducted in daily clinical practice.

In case of a positive evaluation of our intervention, transfer and implementation to the German health care system could be realised in three steps. First, applicable recommendations for performing regular family conferences in patients with frailty and polypharmacy should be added to future clinical guidelines on management of multimorbidity and polypharmacy. Second, German statutory health insurance might consider paying GPs for conducting family conferences in usual care or within the limits of selective contracts. Third, family conferences may be an eligible part of a future disease management programme on multimorbidity and polypharmacy. As this project is funded by the Innovationsfonds of the German Federal Joint Committee aiming only at projects with a high potential for implementation in the German health care system, there is a realistic chance for it, given a positive outcome of the trial.

## Trial status

Patient recruitment opened on 15 March 2019 and is expected to continue to 31 March 2020. Protocol version number 1.1 (15 February 2020).

## Data Availability

Data cannot be made available, owing to the European Union General Data Protection Regulation. Readers who are interested in study materials (e.g., model consent form) should contact the corresponding author.

## Abbreviations

ADR: Adverse drug reaction
AE: Adverse event
CERAD: Consortium to Establish a Registry for Alzheimer’s Disease
COFRAIL: Family Conferences and Shared Prioritisation to Improve Patient Safety in the Frail Elderly
CRF: Crucial structure- and process-related factors
DBI: Drug Burden Index
DSMB: Data and safety monitoring board
GDS: Geriatric Depression Scale
GP: General practitioner
ICER: Incremental cost-effectiveness ratio
ICUR: Incremental cost-utility ratio
NHPP: Number of hospitalisations per patient
PIM: Potentially inappropriate medications
QALY: Quality-adjusted life-year
TNM: Total number of medications
